# A New Departure and a Valuable Analysis

**Published:** 1921-09-10

**Authors:** 


### THE FRACTURE DEPARTMENT.

### A New Departure and a Valuable Analysis.

Among many valuable contributions which appear in the latest number of the Guy's Hospital Reports,* one of particular interest, to both the administrative and surgical staffs of our hospitals, comes from the pen of Mr. E. G. Slesinger, an assistant surgeon and surgeon-in-charge of the fracture department at that hospital.

We agree with the author, that to the best of our knowledge this fracture department, at which all cases of recent fractures in out-patients are treated, is the only one exactly of the kind in London, and it obviously offers unrivalled opportunities for special study of the mechanics, prognosis, and treatment of such simple fractures as can be treated by conservative means. It is very happy, therefore, that Mr. Slesinger has provided so good a summary of the experience already gained.

He deals with a consecutive series of 730 patients attending his department during the last eight months of 1920. In every instance diagnosis was based and confirmed upon *x*-ray plates, and where the functional result was less than 100 per cent. the condition was verified by further *x*-ray examination. On general lines the analysis of these cases shows 458 cases of fractures of the upper limb, or 62.6 per cent., and of these 303 were males and 155 females, but as the author confines his detailed discussion entirely to this one section, we will follow him and hope later to refer to his conclusion in connection with other varieties of fracture. It must be remembered, however, that the percentage incidence does not represent the actual liability of one limb or the other to fracture since a far higher proportion of lower-limb injuries are admitted directly to the wards. The sex incidence must also be viewed as partly a matter of the influence of hospital as opposed to non-institutional practice.

However, let us follow Mr. Slesinger's experience with this particular series of cases. In the first place, it is noteworthy that in the whole lot there was no case of non-union of a fracture. On the other hand, there is evidence that many minor injuries are repeatedly passed over. For example, he says, of fracture of the acromian process in the shoulder region, that it is a singular fact which can only be explained on the assumption that the injury is frequently overlooked, that there was only one example of this fracture noted. Yet it is by no means uncommon in the ordinary surgical out-patients to see people presenting obvious evidence of an old fracture in this region, and who are nevertheless unaware of ever having sustained a fracture at the time of their original injury.

### Fractures of the Clavicle.

Again, so well shown a mishap as fracture of the collar bone or clavicle is worth noting in its statistical form. This type formed 16.8 per cent. of the upper-limb fractures, and nearly a fifth of them were green-stick—*i.e.*, incomplete fractures, and no less than 79 per cent. occurred at ages less than twenty years. Male cases were forty-six, against thirty-one in females, indicating, in view of the general sex incidence (2 to 1) already noted, that females are decidedly more prone to fracture of this bone. Almost invariably the site of breakage was in one of two situations, either the outer third of the bone from violence or the middle third from indirect stress, and as the author is herein at variance with the majority of text-books we quote him fully.

The usual description in surgical text-books of this latter fracture is that it occurs at the junction of the two curves of the bone. This I believe to be incorrect in fact, as well as being mechanically unlikely; the usual situation of this fracture, in our experience, is well within the anterior concavity. The displacement is fairly constant; the fractured ends are displaced upwards and forwards, the shoulder and with it the outer fragment is dropped and displaced forwards, and the outer end of the inner fragment usually overlaps the outer fragment. The displacement is usually described as being brought about by muscular action, a statement constantly made in reference to displacement in fractures, and for which I would maintain there is, in the majority of cases, not the least evidence. There can, I think, be little reasonable doubt that the displacing force in a fracture is the force which causes the fracture, and every evidence points to the action of muscles as being quite negligible in this respect. To know the mechanism by which a particular fracture is produced is to know the displacement, and the degree of such displacement will vary with the fracturing force and the mechanical advantage at which it acts.

In any case the treatment of all such cases appears to be remarkably satisfactory from a functional point of view, even though it is impossible always to obtain perfect anatomical reposition. In the case of the clavicle the author considers this due to the fact that the muscles do not act along an axis parallel to its length, and that therefore small alterations in length or curvature of the bone do not effect mechanical efficiency to the same extent as with the majority of long bones.

### Fractures of the Upper Arm.

Breakage of the humerus, including separation of the epiphyses, formed only 15.2 per cent. of the upper-limb cases. The smallness of this figure is a little surprising when drawn from hospital practice, but with regard to the work performed upon these cases it is to be remembered that in a large proportion of fractures of the shaft of the humerus manipulative reduction is so imperfect that the open operation gives better results. The same applies to separated epiphyses and all T-shaped fractures into the joint at the lower end, and it is obvious, therefore, that many fractures of this bone pass beyond

* Published by Henry Frowde and Hodder & Stoughton.

the scope of out-patient treatment. In any case Mr. Slesinger is emphatic that with all humerus cases, and, indeed, all in the upper limb, the proper position is one in which all the gravity-opposing muscles are kept short at the expense of the gravity-aided opponents. Abduction and elevation at the shoulder, flexion at the elbow, supination in the forearm, dorsiflexion at the wrist, and separation of the fingers are all *anti*-gravity movements. If, during the process of repair, these muscles are kept short, the opposite movements will be readily restored on releasing the limb by the action of gravity, aided, if need be, by simply giving the patient a weight attached to the hand. It is a simple principle and one which will, we think, be readily appreciated and understood.

### The Lower Arm.

The detailed discussion of fractures of the radius and ulna is here forbidden by lack of space, but we note a number of interesting points from this series. The radius alone was fractured in 151 cases, and with the ulna in 234—*i.e.*, the radius is by far the commonest site of arm injury. When the fracture occurs through the neck of the radius, and there is much displacement of the head, the author, from his experience, favours the immediate removal of the upper fragment as giving better functional results. The series shows that Colles' fracture is markedly more frequent in women (four times), and the ages of the patients are interesting in that the average for women was 53.2 years and considerably higher than in men, who gave forty-two years.

Concerning treatment, the author strongly advocates reduction of a Colles' fracture over a rectangular splint, well padded, and with the wrist so fixed with strapping that for forty-eight hours there is complete flexion to a right angle. After this the splint is removed and gentle massage given, and the forearm put on a slightly cock-up splint, with a tennis ball in the hand. Daily massage follows, and the patient is encouraged to move the fingers by gripping the ball frequently. The use of whirlpool baths before massage in these cases is strongly advocated. The average length of treatment in females was 5.9 weeks and in males 4.5 weeks.

In the so-called Chauffeur's fracture displacement was invariably found to be slight, and the cases were all treated by immediate massage, with whirlpool baths, and were rested in a sling between treatments. With an average of three weeks all gave perfect results.

There were thirty-four cases of complete fracture of radius and ulna, males showing a marked preponderance, and the age distribution tending markedly to early life. Mr. Slesinger holds that there is but one way of handling these cases if a good functional result is to be obtained—namely, by treating them in full supination. In any case, supination is certainly of more physiological importance than pronation, and the author's method is in full accord with his anti-gravity principle as already noted.

Also an interesting method is outlined for obtaining the great extension necessary in very oblique fractures of the finger bones. The forearm and hand, with a tennis ball grasped, are fixed upon a straight splint by strapping. A hole is bored (this is quite painless) in the free edge of the nail, and a salmon-gut and rubber extension band fixed to a peg at the end of the splint. It is a simple, but a remarkably efficient method, allowing that slight movement of the joints which is invaluable in the avoidance of stiffness. Here, again, all Mr. Slesinger's cases gave 100 per cent. after-function, and we congratulate him, not only upon the general interest of his paper, but the perfection to which systematic fracture work in an out-patient department can be brought.

